# A novel lncRNA-hidden polypeptide regulates malignant phenotypes and pemetrexed sensitivity in A549 pulmonary adenocarcinoma cells

**DOI:** 10.1007/s00726-023-03361-7

**Published:** 2024-02-13

**Authors:** Xiaobing Han, Liangxin Chen, Peng Sun, Xiuqing Wang, Qian Zhao, Lingfeng Liao, Dejin Lou, Nan Zhou, Yujun Wang

**Affiliations:** 1 Department of Oncology, Xinyang Central Hospital, No. 1 Siyi Road, Shihe District, Xinyang, 464000 Henan China; 2 Department of Gastroenterology, Xinyang Central Hospital, No. 1 Siyi Road, Shihe District, Xinyang, 464000 Henan China

**Keywords:** NSCLC, lncRNA-hidden polypeptides, Short ORFs, LINC00954-ORF, PEM resistance

## Abstract

The advance of high-throughput sequencing enhances the discovery of short ORFs embedded in long non-coding RNAs (lncRNAs). Here, we uncovered the production and biological activity of lncRNA-hidden polypeptides in lung adenocarcinoma (LUAD). In the present study, bioinformatics was used to screen the lncRNA-hidden polypeptides in LUAD. Analysis of protein expression was done by western blot or immunofluorescence assay. The functions of the polypeptide were determined by detecting its effects on cell viability, proliferation, migration, invasion, and pemetrexed (PEM) sensitivity. The protein interactors of the polypeptide were analyzed by mass spectrometry after Co-immunoprecipitation (Co-IP) assay. The results showed that the lncRNA LINC00954 was confirmed to encode a novel polypeptide LINC00954-ORF. The polypeptide had tumor-suppressor features in A549 cells by repressing cell growth, motility and invasion. Moreover, the polypeptide enhanced PEM sensitivity and suppressed growth in A549/PEM cells. The protein interactors of this polypeptide had close correlations with RNA processing, amide metabolic process, translation, RNA binding, RNA transport, and DNA replication. As a conclusion, the LINC00954-ORF polypeptide embedded in lncRNA LINC00954 possesses tumor-suppressor features in A549 and PEM-resistant A549 cells and sensitizes PEM-resistant A549 cells to PEM, providing evidence that the LINC00954-ORF polypeptide is a potential anti-cancer agent in LUAD.

**Supplementary Information:**

The online version contains supplementary material available at 10.1007/s00726-023-03361-7.

## Introduction

As the most prevalent oncological disease, pulmonary cancer causes almost 1.8 million deaths every year all over the world. In 2020, pulmonary cancer is responsible for 11.4% of global cancer morbidity and 18.0% of cancer mortality (Sung et al. 2020). Non-small cell lung cancer (NSCLC), the more common subtype of pulmonary cancer, divides into non-squamous and squamous types according to histopathological features (Imyanitov et al. 2021). In Asian countries including China, non-squamous NSCLC constitutes the majority of all pulmonary cancer cases, and lung adenocarcinoma (LUAD) is the predominant NSCLC entity (Chen et al. 2022; Warkentin et al. 2022). In the last decade, innovatory advances have been achieved in NSCLC therapeutic regimens, such as immunotherapy and chemo-immunotherapy combination, thereby leading to an improved survival in advanced NSCLC (Jasper et al. 2022). Despite these efforts, the survival rate of NSCLC remains persistently low. Meantime, resistance of chemo-therapeutic drugs frequently occurs (Wu and Lin 2022). For these reasons, expansion of treatment regimens is essential for the improvement of this disease outcome.

Owing to the lack of long or conserved ORFs, long non-coding RNAs (lncRNAs) were initially regarded as RNA transcripts that cannot encode proteins (Bridges et al. 2021; Herman et al. 2022). Recently, the advance of high-throughput sequencing enhances the discovery of short ORFs embedded in lncRNAs. Some of these short ORFs have been demonstrated to able to encode polypeptides of less than 100 amino acids (aa) (Zhang et al. 2021; Xing et al. 2020). Despite the small size, these lncRNA-derived polypeptides are frequently biologically relevant in that they are essential for diverse pathological processes, including oncological disease (Zhou et al. 2021; Wu et al. 2020). Recent work shows a notion that the anti-cancer property of lncRNA-hidden immunogenic polypeptides may operate as a tumor vaccine (Barczak et al. 2023). Furthermore, several hidden polypeptides derived from lncRNAs have been uncovered as onco-peptides (such as polypeptide ASAP hidden in LINC00467) or anti-cancer agents (such as LINC00665-encoded micropeptide CIP2A-BP) in carcinogenesis (Guo et al. 2020; Ge et al. 2021). Therefore, it would to intriguing to uncover the production and biological activity of such polypeptides for cancer management.

Here, we first found out putative lncRNA-hidden polypeptides in LUAD through bioinformatics. Subsequently, we confirmed a true lncRNA-derived polypeptide and revealed its functions in LUAD tumorigenesis and pemetrexed (PEM) resistance with the A549 LUAD cell line. Finally, we decided to preliminarily explore target proteins affected by the polypeptide via mass spectrometry analysis after Co-immunoprecipitation (Co-IP) experiment.

## Materials and methods

### Bioinformatics

RNA-seq data of TCGA-LUAD and the corresponding clinical features were retrieved from TCGA database (https://portal.gdc.cancer.gov). LncRNAs that are related to LUAD prognosis were searched by R survival package and differential expression analysis was done using DESeq2 (Love et al. 2014). The diagnostic potential was evaluated by receiver operating characteristic (ROC) curves using pROC package in R. Correlation analysis of the lncRNA LINC00954 and chemoresistance-related factors was done using Pearson’s correlation analysis by R ggplot2. For biological function analysis of the protein interactors, GO and KEGG enrichment analyses were done using the online DAVID database (https://david.ncifcrf.gov/home.jsp).

### Cell lines

Under standard conditions (37 ℃, 5% cardon dioxide), A549 LUAD cells (#CL-0016, Procell, Wuhan, China) were cultivated in 10% FBS Ham’s F-12 K (#CM-0016, Procell). For function analysis of the polypeptide in pemetrexed (PEM) resistance, we generated the PEM-resistant A549 (A549/PEM) LUAD cell line in a stepwise manner as described (Hsin et al. 2020). Briefly, the A549 parental cell line was stimulated with increasing concentrations (50 nM initial concentration with increasing in multiples of two) of PEM (Selleck, Shanghai, China). The development of the PEM-resistant cell line was determined by calculating the PEM-resistant index (RI) using the formula: RI = the IC50 value of A549/PEM cells/the IC50 value of A549 cells. The A549/PEM cells were identified to have the phenotype of PEM resistance when RI > 5. For resistance maintenance, 10% FBS Ham’s F-12 K containing 0.5 μM of PEM was used for cultivation of A549/PEM cells. For evaluation of PEM efficacy, A549/PEM cells were subjected to PEM stimulation at 5 μM for 24 h.

### Expression constructs and transfection

To confirm the coding ability of the ORFs embedded in lncRNAs, we cloned these ORFs including LINC00954-ORF into the pcDNA3.1–3 × FLAG basic vector (Miaoling Biology, Wuhan, China), respectively, based on HindIII and XhoI restriction sites. The mutant construct (LINC00954-ORFmut) was generated by ligating the mutant LINC00954-ORF sequence, in which the start codon ATG was mutated to ATT, into the pcDNA3.1–3 × FLAG vector. To express the lncRNA LINC00954 in A549 cells, the pcDNA3.1-LINC00954 construct was produced by subcloning the LINC00954 sequence into the basic vector. All sequences were made by Tsingke Biotech (Beijing, China).

A549 and A549/PEM LUAD cells were seeded into culture plates on the day prior to transfection. The relevant expression construct was transfected as described by the manufacturer (Thermo Fisher Scientific, Saint-Aubin, France), using Lipofectamine 3000. Cells were subjected to further analyses 48 h post transfection.

### Generation of antibody to LINC00954-ORF polypeptide

The polyclonal antibody to LINC00954-ORF polypeptide was made by Writegene (Zhengzhou, China). Briefly, after production of this polypeptide, the antibody was made by inoculating the polypeptide into rabbits and collecting the blood samples before affinity chromatography for purification.

### Protein analysis by western blot

After expression construct introduction or/and PEM stimulation, the cells were used for protein extraction, and followed by quantitation of the protein content by BCA assay and protocols (Thermo Fisher Scientific). For immunoblotting, after SDS-PAGE, the resulting gels were processed by electro-transferring to nitrocellulose (Millipore, Darmstadt, Germany), which were subsequently probed with anti-rabbit LINC00954-ORF polypeptide antibody (1:500), anti-rabbit Flag tag antibody (#GB11938, Servicebio, Wuhan, China, 1:800), anti-mouse PCNA antibody (#60097–1-Ig, Proteintech, Wuhan, China, 1:20000), or anti-rabbit CDK1 antibody (#10762–1-AP, Proteintech, 1:3000). Equal loading of the protein extract was standardized with anti-mouse β-actin antibody (#GB15001, Servicebio) at a 1:1500 dilution. The HRP-linked anti-rabbit (#ab6721, 1:5000) or anti-mouse (#205719, 1:15000) secondary antibody was procured from Abcam (Shanghai, China). For blot development, a hyper-sensitive ECL Kit was applied as per the accompanying protocols (Beyotime, Shanghai, China).

### Immunofluorescence microscopy

A549 LUAD cells after transfection by LINC00954 expression plasmid, LINC00954-ORF construct, or control mock were subjected to fixation, permeabilization (0.5% Triton X-100), and blocking (3% BSA). Antibodies to LINC00954-ORF polypeptide (1:300), PCNA (#60097–1-Ig, 1:500) and MMP9 (#ab76003, Abcam, Cambridge, UK, 1:250) were diluted in PBS and added to the cells, respectively, and followed by incubation overnight at 4 ℃. Secondary antibodies were procured from Servicebio, including anti-rabbit (#GB25303, 1:600) or anti-mouse (#GB25301, 1:800) IgG cross-adsorbed Alexa Fluor 488. Nuclear staining was done by DAPI incubation. Through Olympus CKX53 microscopy (Olympus, Tokyo, Japan), images were acquired for data analysis.

### CCK-8 viability and cytotoxicity assays

The in vitro viability and cytotoxicity were determined by CCK-8 assay and protocols (Beyotime). In viability assay, after A549 and A549/PEM LUAD cells were subjected to the relevant transfection or/and PEM exposure, CCK-8 reagent was added to the cells, followed by incubation for 2 h at 37 ℃. In cytotoxicity assay, un-transfected or transfected LUAD cells were challenged with various concentrations of PEM for 24 h. After that, CCK-8 reagent was used as described. Plates were read using a Thermo MULTISKAN MK3 plate reader at 450 nm. Based on the plot of the percentage of viable cells relative to PEM concentration, we scored the IC50 value.

### Calcein-AM/PI staining

For this staining, the Calcein-AM/PI double staining kit was applied as described by the Servicebio. In brief, after the relevant transfection, the cells were re-suspended in working buffer of Calcein AM and PI, and followed by a 20 min incubation at 37 ℃. Microscopic fluorescence images were acquired for data analysis (the ratio of Green/Green + Red).

### EdU proliferation assay

The impacts of the LINC00954-ORF polypeptide and combination of the peptide and PEM on the growth of A549 and A549/PEM LUAD cells were evaluated by EdU assay using Click-iT EdU-555 Proliferation Kit from Servicebio. In brief, after the cells were treated as indicated, EdU working reagent was added to the cells. After 2 h, the Hoechst 33342 was diluted in PBS and added to the cells, which was followed by a 5 min incubation. Microscopic fluorescence images were acquired at least five random fields.

### Wound-healing assay

To evaluate the influence of the LINC00954-ORF polypeptide in cell motility, the assay was done as reported by Qiu et al*.* (Qiu et al. 2019). LINC00954-ORF construct- or pcDNA-NC-transfected A549 cells were plated onto 6-well plates. A vertical wound was subsequently produced by a sterile forcep. After 24 h of cultivation at 37 ℃, we acquired the microscopic images of wound closure.

### Transwell invasion assay

Analysis of the effect of the LINC00954-ORF polypeptide on invasion was done using Transwell Invasion Matrigel^®^ chamber (Corning, Shanghai, China). LINC00954-ORF construct- or pcDNA-NC-transfected A549 cells were placed on insert membranes in non-serum media and were allowed to traverse to 10% FBS Ham’s F-12 K in the lower compartment. One day later, invasive cells were scored after image acquisition.

### Co-immunoprecipitation (Co-IP) assay

Co-IP experiment was done as described (Zheng et al. 2023) using 10^7^ LINC00954-ORF construct-transfected A549 cells. Briefly, proteins extracted from the cells were immunoprecipitated and purified with anti-Flag tag affinity Sepharose gels or anti-IgG controls (Yeasen, Shanghai, China). Beads were washed, and the co-precipitated proteins were harvested with the eluent (Beyotime). A silver staining method after SDS-PAGE was subsequently used for the visualization of the co-precipitated proteins with Protein Silver Staining Kit provided by Servicebio.

### Mass spectrometry analysis

The co-precipitated proteins in Co-IP assay were subjected to mass spectrometry analysis using the L-3000 HPLC System (RIGOL, Beijing, China) after desalination. Raw mass spectrometry data were processed by Proteome Discoverer2.4 (Thermo Fisher Scientific).

### Statistical analysis

In figures, data were presented as mean ± SD of three biological repetitions. Unpaired two-tailed *t* test was applied for analysis of two groups. For multiple group analysis, one-way ANOVA was utilized. Significance was set to *P* value less than 0.05.

## Results

### Screen of lncRNA-hidden polypeptides in LUAD

**Fig. 1:**
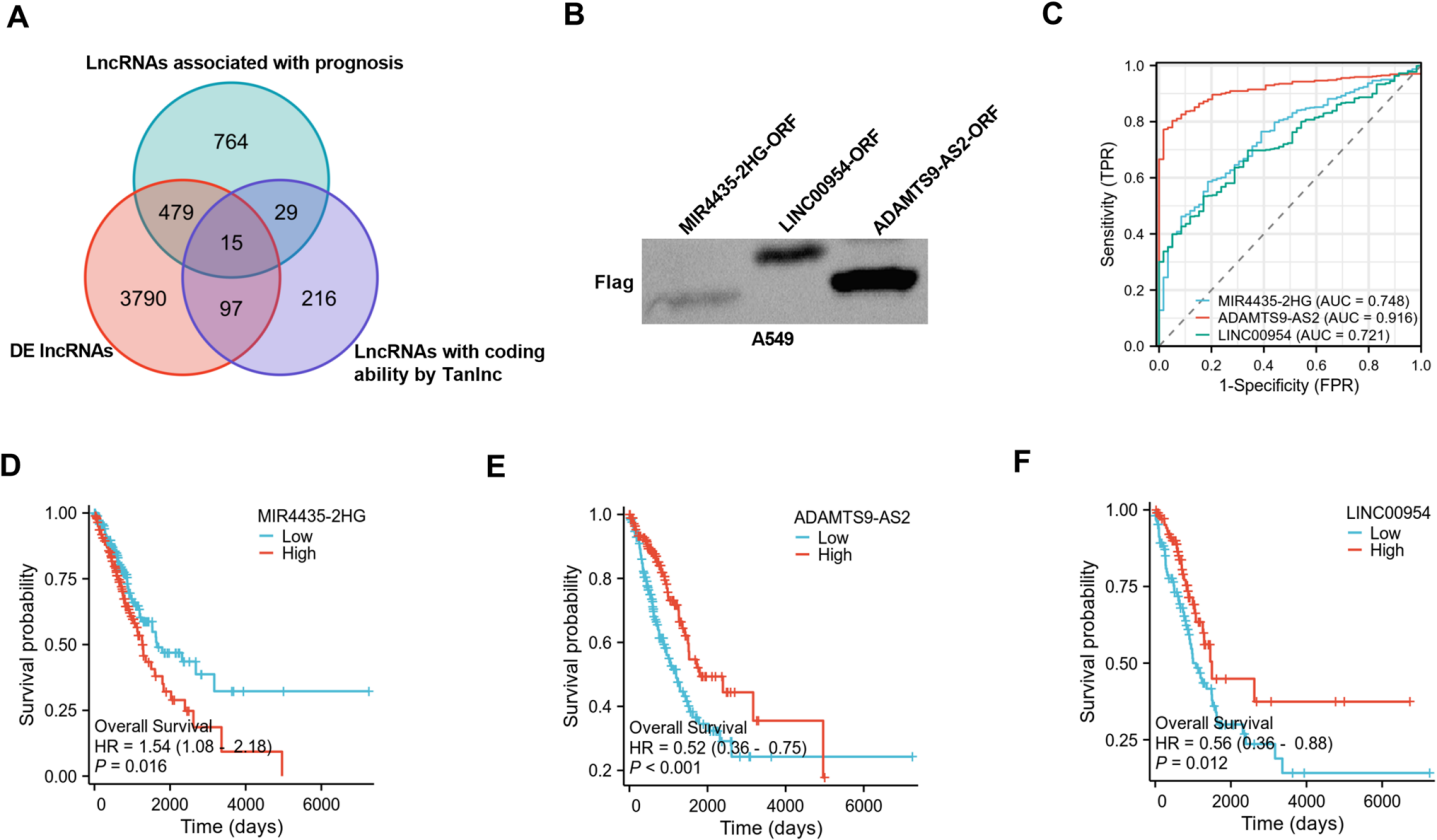
Screen of polypeptides hidden in lncRNAs in LUAD. (**A**) Venn diagram of the screening method and the 15 candidates. (**B**) Expression of MIR44435-2HG-ORF, ADAMTS9-AS2-ORF and LINC00954-ORF polypeptides demonstrated by western blot. (**C**) pROC package in R predicted the diagnostic potential of MIR44435-2HG, ADAMTS9-AS2 and LINC00954 in LUAD using TCGA-LUAD dataset. (**D**–**F**) Survival package of R software analyzed the prognosis correlation of MIR44435-2HG, ADAMTS9-AS2 and LINC00954 expression in LUAD

In order to explore the lncRNAs-encoded polypeptides in LUAD, first the RNA-seq data of TCGA-LUAD and corresponding clinical features were retrieved from TCGA database (https://portal.gdc.cancer.gov). The data were systematically mined to find out 1287 lncRNAs related to LUAD prognosis by the survival package of R software (Supplementary Table 1) and 4381 differentially expressed (DE) lncRNAs by DESeq2 (|log(Fold Change)|> = 1 and *P* < 0.05) (Supplementary Table 2). Second, using online database Tranlnc (http://bio-bigdata.hrbmu.edu.cn/TransLnc/index.jsp), 357 lncRNAs with coding ability (their mass spectrometry data and occupancy of ribosomes had been confirmed) were found. Finally, Venn diagram was used to visualize the common elements shared by the above three categories. As illustrated in Fig. 1A, in total 15 lncRNAs that are related to LUAD prognosis, show aberrant expression in LUAD, and possess potential-coding potential were found (Supplementary Table 3).

The expression analyses of these candidates by western blot in A549 cells demonstrated the coding ability of the three lncRNAs (MIR44435-2HG, ADAMTS9-AS2 and LINC00954) (Fig. 1B) when the respective small ORFs were subcloned in the pcDNA3.1-basic vector fused with 3 × Flag tags. Interestingly, ROC curves by pROC package in R showed the high diagnostic potential of the three lncRNAs in LUAD (Fig. 1C). Moreover, high levels of MIR4435-2HG predicted poor prognosis of LUAD patients (Fig. 1D); ADAMTS9-AS2 expression was positively associated with overall survival of patients with LUAD within 4000 days of post-operative follow-up (Fig. 1E); patients with high LINC00954 expression had better outcomes than those with low LINC00954 expression (Fig. 1F). In addition, LINC00954 expression had significant associations with pathologic T stage, N stage and pathologic stage of LUAD tumors (Supplementary Table 4). We, therefore, focused on LINC00954-hidden polypeptides in this work.

### LINC00954 encodes a novel polypeptide LINC00954-ORF

**Fig. 2:**
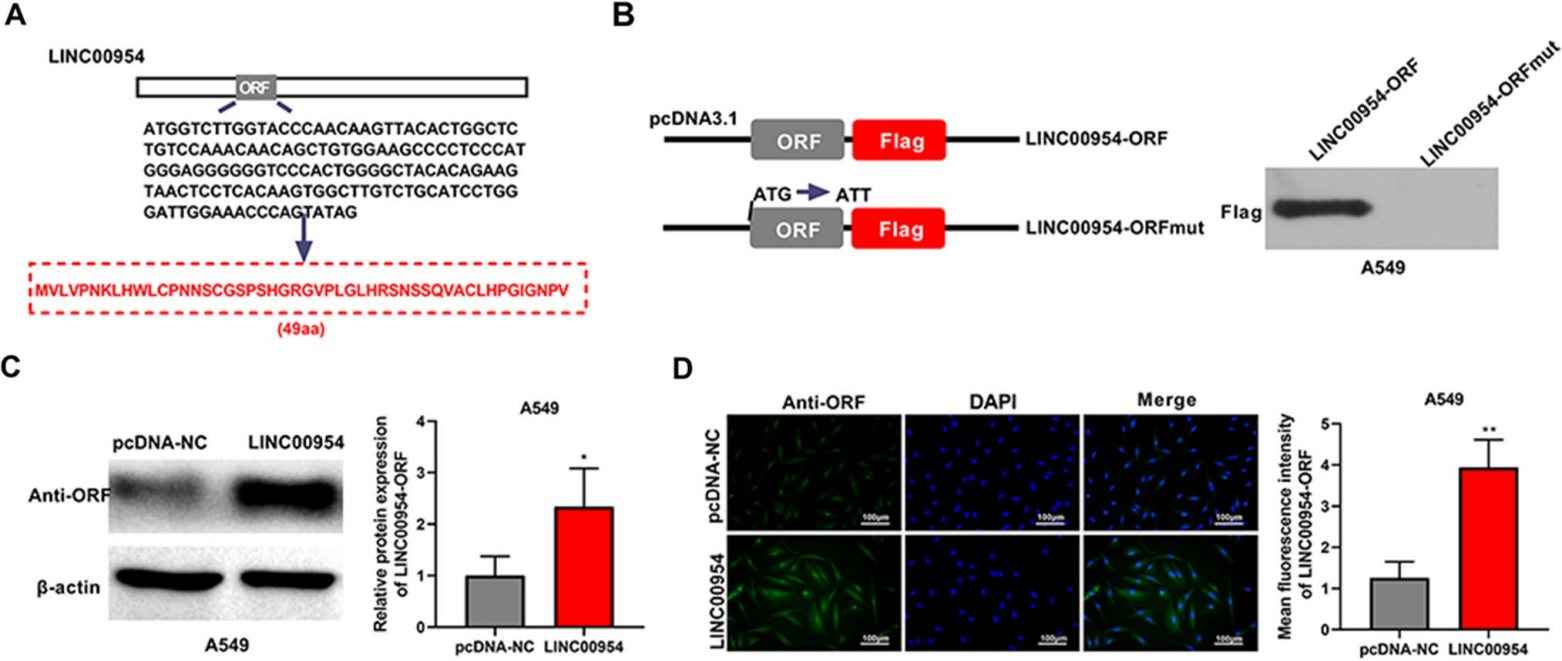
The lncRNA LINC00954 encodes a 49-aa polypeptide in A549 cells. (**A**) Diagram of the LINC00954 ORF, with the sequence of its hidden polypeptide. (**B**) Left: Schematic representation of the wild type (LINC00954-ORF) and mutant (LINC00954-ORFmut) constructs. In LINC00954-ORFmut plasmid, the ATG start codon was mutated to ATT. Right: Western blot detected the expression of the LINC00954-ORF-Flag polypeptide in A549 cells transfected as indicated. (**C** and **D**) Western blot (**C**) and immunofluorescence (**D**) showed the level of LINC00954-ORF polypeptide in A549 LUAD cells transfected with pcDNA-NC control vector or LINC00954 expression plasmid. *P < 0.05, **P < 0.01

The LINC00954, located in chr2:19875983–19876129[ +], harbors a 150-bp small ORF with the ATG start codon and the TAG end codon, which can encode a 49-aa polypeptide (Fig. 2A). Through a mutant vector (LINC00954-ORFmut) in which the ATG start codon is mutated to ATT (Fig. 2B), the biological activity of the start codon was confirmed by western blot, as shown by the evidence that no polypeptides fused with Flag tags were detected in A549 cells after introduction of LINC00954-ORFmut vector (Fig. 2B).

To confirm the protein-coding ability of LINC00954, we also generated a plasmid construct expressing LINC00954 and transfected it into A549 cells. Meanwhile, a rabbit polyclonal antibody to LINC00954-ORF polypeptide was produced. By western blot and immunofluorescence with the specific antibody, the endogenous existence of the polypeptide in A549 cells was demonstrated (Fig. 2C). Furthermore, LINC00954 expression construct-introduced A549 cells exhibited high levels of the LINC00954-ORF polypeptide than pcDNA-NC controls (Fig. 2D), reinforcing the protein-coding capacity of lncRNA LINC00954.

### Tumor-suppressor features of LINC00954-ORF polypeptide in A549 cells

**Fig. 3:**
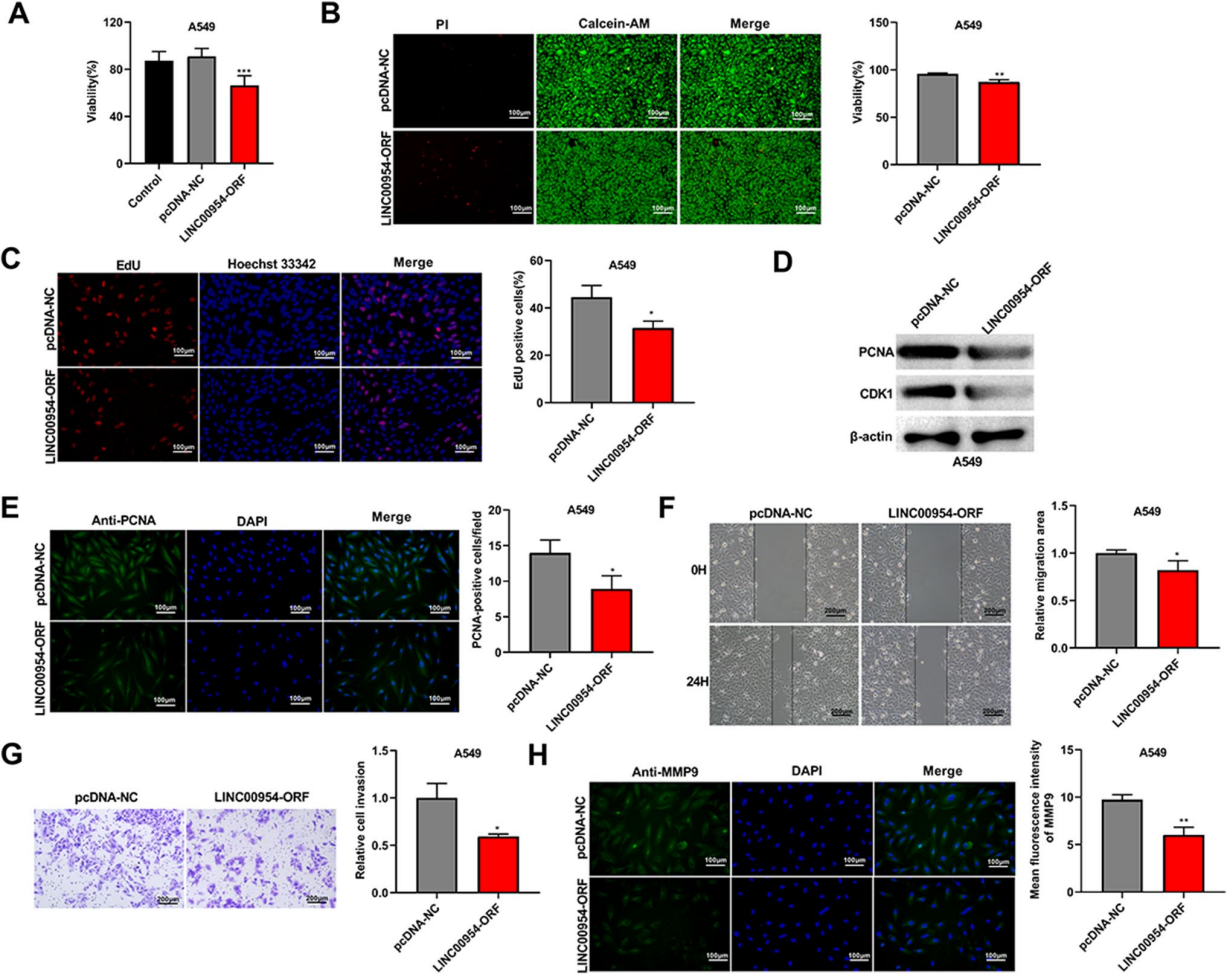
LINC00954-ORF polypeptide exerts suppressive functions in malignant phenotypes of A549 cells. (**A** and **B**) CCK-8 assay and Calcein-AM/PI staining showed that A549 cells with upregulation of LINC00954-ORF polypeptide were less viable than pcDNA-NC control vector-transfected cells. (**C**) EdU assay displayed the reduction of the EdU-positive cells in LINC00954-ORF introduction group compared with control vector group. (**D**) Western blot detected the downregulation of PCNA and CDK1 in LINC00954-ORF transfected cells. (**E**) Immunofluorescence showed the reduced PCNA expression in LINC00954-ORF transfected cells. (**F**) Wound-healing assay confirmed the repressive function of LINC00954-ORF polypeptide in A549 cell motility. (**G**) Transwell assay showed the suppressed invasion rate in LINC00954-ORF transfected cells compared with control vector introduced cells. (**H**) Immunofluorescence revealed the reduced expression of MMP9 in LINC00954-ORF transfected cells. *P < 0.05, **P < 0.01, ***P < 0.001

Having demonstrated the existence of the LINC00954-ORF polypeptide in A549 cells, we wished to elucidate its contribution to the tumorigenic phenotype. Thus, we upregulated this polypeptide by LINC00954-ORF plasmid introduction to show if this polypeptide causes alterations in cell growth, migratory activity, and invasiveness. Remarkably, the increase of the LINC00954-ORF polypeptide in A549 cells had tumor growth-inhibitor features (Fig. 3A–C). Upon LINC00954-ORF plasmid introduction, the cells showed less viable in the CCK-8 assay (Fig. 3A) and Calcein-AM/PI staining (the reduced ratio of Green/Green + Red, Fig. 3B) and had significantly decreased EdU-positive cells (Fig. 3C). This impairment of this polypeptide in cell growth was partially associated with the downregulation of PCNA and CDK1 (Fig. 3D and E), two essential regulators in cell cycle progression. We next tested the ability of the LINC00954-ORF polypeptide to influence cell migratory capacity and invasiveness of A549 cells compared with negative vector-transfected cells. In contrast, the cells with upregulation of this polypeptide showed repressive migration and invasion rates (Fig. 3F and G). To reinforce its repression effects on cell motility and invasiveness, we carried out immunofluorescence for the related MMP9 protein and found that in LINC00954-ORF-introduced cells, the MMP9 protein expression was downregulated (Fig. 3H).

### LINC00954-ORF polypeptide enhances PEM sensitivity and suppresses growth in A549/PEM cells

**Fig. 4:**
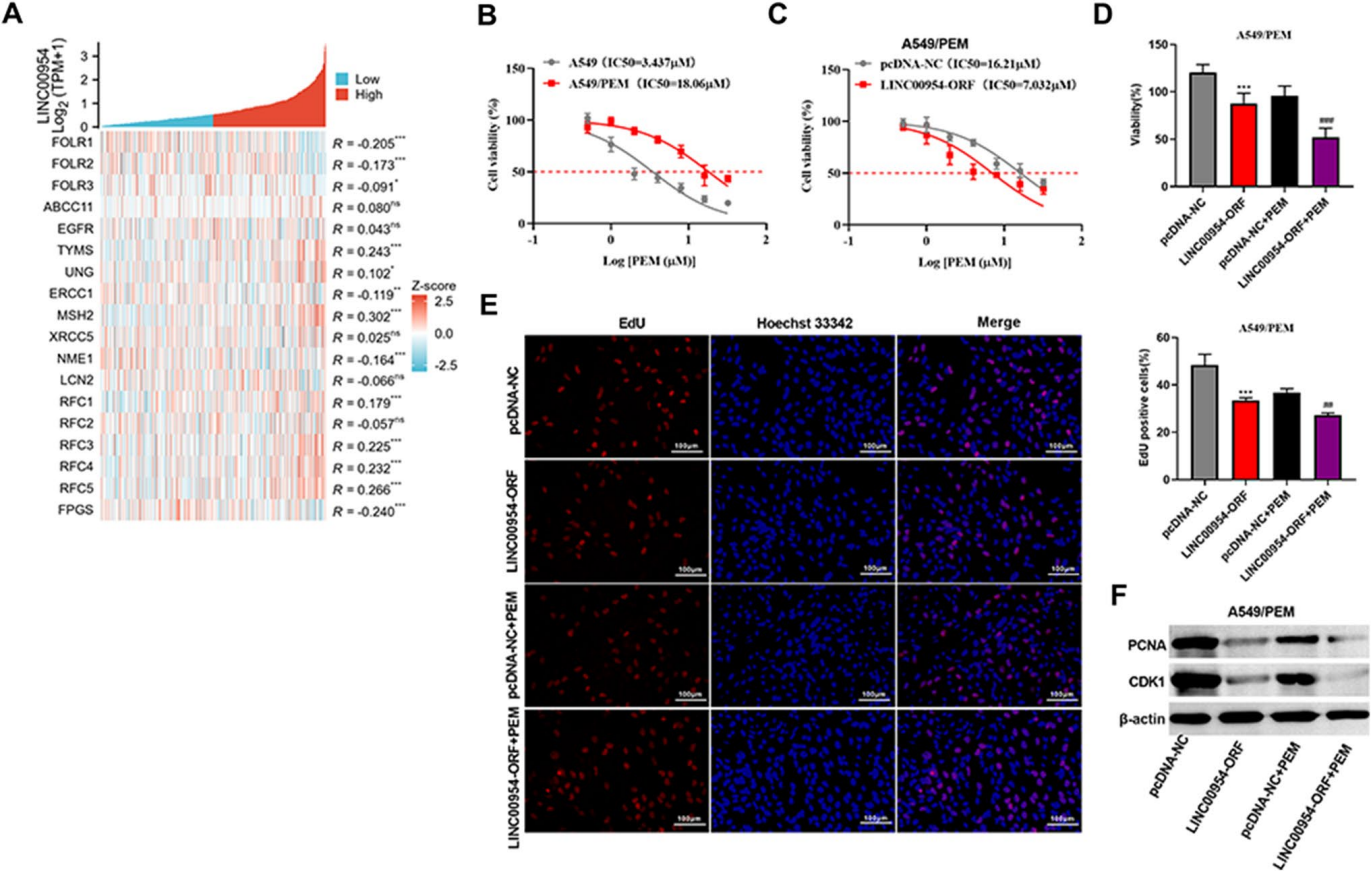
LINC00954-ORF polypeptide enhances PEM sensitivity and suppresses growth in A549/PEM cells. (**A**) Pearson’s correlation analysis of lncRNA LINC00954 and the chemoresistance-related genes in TCGA-LUAD. (**B**) Cell viability and IC50 value for PEM of A549 and A549/PEM cells. (**C**) Cell viability and IC50 value for PEM of A549/PEM cells after transfection by pcDNA-NC control vector or LINC00954-ORF plasmid. (**D**) Viability of A549/PEM cells after the indicated transfection and treatment with or without PEM. (**E**) Proliferation evaluation of A549/PEM cells after the indicated transfection and treatment with or without PEM. (**F**) Western blot of PCNA and CDK1 in A549/PEM cells treated as indicated. ***P < 0.001, ^##^*P* < 0.01, ^###^*P* < 0.001

Using Pearson’s correlation analysis in TCGA-LUAD, heat map by R ggplot2 showed that the lncRNA LINC00954 had close association with some chemoresistance-related factors, such as FOLR1, TYMS and FPGS (Fig. 4A), suggesting the implication of LINC00954 in drug resistance. We thus wondered whether the LINC00954-ORF polypeptide has an impact on PEM resistance of LUAD. To address this issue, we established a PEM-resistant A549 cell line (A549/PEM). By the CCK-8 assay, A549/PEM cells (IC50 = 18.06 μM) had a higher value of IC50 for PEM than A549 parental cells (IC50 = 3.437 μM) (Fig. 4B), demonstrating the successful establishment of PEM-resistant cells. Importantly, the LINC00954-ORF polypeptide sensitized A549/PEM cells to PEM therapy (Fig. 4C). Upon the transfection of LINC00954-ORF plasmid, the resistant cells (IC50 = 7.032 μM) exhibited a lesser value of IC50 than control vector-transfected cells (IC50 = 16.21 μM) (Fig. 4C). We also determined the ability of this polypeptide to regulate the growth of A549/PEM cells. Consistent with A549 parental cells, we also observed the growth-suppressive activity of the LINC00954-ORF polypeptide in A549/PEM cells (Fig. 4D–F). Under administration with or without PEM, A549/PEM cells with this polypeptide upregulation showed less viable in the CCK-8 assay (Fig. 4D), had significantly reduction in the EdU-positive cells (Fig. 4E), as well as exhibited downregulated expression of PCNA and CDK1 (Fig. 4F) compared with their counterparts.

### Analysis of the interacted proteins of LINC00954-ORF polypeptide

**Fig. 5:**
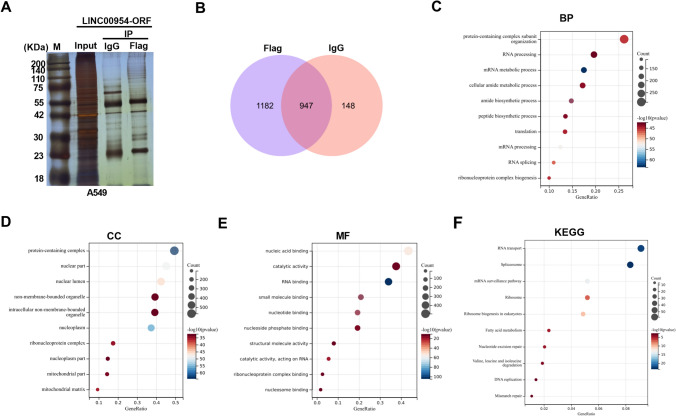
Analysis of the target proteins of LINC00954-ORF polypeptide. (**A**) Confirmation of Co-IP experiment results by a silver staining method. (**B**) Schematic of the screen of the 1182 unique proteins in the Flag group. (**C**) The biological processes (BP) enriched by the 1182 unique proteins in the Flag group. (**D**) The cellular components (CC) enriched by the 1182 unique proteins in the Flag group. (**E**) The molecular function (MF) enriched by the 1182 unique proteins in the Flag group. (**F**) The KEGG pathways enriched by the 1182 unique proteins in the Flag group

To preliminarily observe target proteins affected by the LINC00954-ORF polypeptide, we carried out mass spectrometry analysis after Co-IP experiment with anti-Flag Sepharose beads or anti-IgG controls. Silver staining results showed a specific band in the Flag group relative to the IgG control group (Fig. 5A). By analyzing the mass spectrometry data, 1182 unique proteins in the Flag group were identified after excluding 947 proteins that were also found in the IgG group (Fig. 5B and Supplementary Table 5), indicating that a total of 1182 proteins that interact with LINC00954-ORF polypeptide were identified. We then analyzed the biological function of these unique proteins by performing GO and KEGG enrichment analyses using DAVID database (https://david.ncifcrf.gov/home.jsp). For the analysis of biological processes (BP), these proteins were markedly associated with protein-containing complex subunit organization, RNA processing, amide metabolic process, translation, and so on (Fig. 5C). In terms of cellular components (CC), these proteins had close correlations with various CC, including protein-containing complex, nuclear lumen, mitochondrial part and mitochondrial matrix (Fig. 5D). For molecular function (MF) analysis, these proteins were observed to be significantly related to nucleic acid binding, catalytic activity, RNA binding, nucleoside phosphate binding, and so on (Fig. 5E). Furthermore, for KEGG pathway analysis, some pathways, such as RNA transport and DNA replication, were enriched (Fig. 5F).

## Discussion

With the development of high-throughput sequencing, a growing amount of short ORFs hidden in lncRNAs are demonstrated to encode functional polypeptides (Zhang et al. 2021). Nonetheless, our understanding of the production, biology and potential of these polypeptides remains in its infancy. Here, we report a novel LINC00954-derived polypeptide LINC00954-ORF that possesses tumor-suppressor features in A549 and A549/PEM LUAD cells. Furthermore, we uncover its promoting activity in PEM sensitivity of A549/PEM cells. These exciting findings illustrate the dual role of such polypeptide in not only directly working as a potential therapeutic agent for LUAD but also enhancing PEM efficacy in LUAD management.

These recent findings have uncovered the therapeutic values of some polypeptides embedded in lncRNAs in oncological disease. As an example, the polypeptide hidden in lncRNA KDM4A-AS1 leads to suppressed viability and reduced migratory capacity in esophageal squamous cell carcinoma, which also modulated fatty acid metabolism (Zhou et al. 2023). LINC00665-drived polypeptide has tumor-suppressor features in osteosarcoma cells through repression of motility and proliferation by distracting the interaction of CREB1 and RPS6KA3 (Pan et al. 2023). Li and colleagues uncovered a novel lncRNA-encoded polypeptide, termed micropeptide MIAC, which weakens cancer cell growth and metastasis and accelerates apoptosis in renal cell carcinoma by inactivating the pathway of EREG/EGFR (Li et al. 2022). In hepatocellular carcinoma, Xiang and colleagues demonstrated the cancer-inhibitory activity of PINT87aa embedded in lncRNA LINC-PINT depending on its suppression of FOXM1-mediated PHB2 (Xiang et al. 2021). Furthermore, the polypeptide pep-AP hidden in lnc-AP can enhance the efficacy of oxaliplatin in colorectal cancer (Wang et al. 2022). In this study, we uncover an uncharacterized polypeptide LINC00954-ORF that is encoded by the prematurely baptized lncRNA LINC00954, a potential biomarker for aneurysmal subarachnoid hemorrhage predicted by bioinformatics (Cheng et al. 2022). By elevating the expression of lncRNA LINC00954 in A549 cells, we further demonstrate the upregulation of this polypeptide in the cell line, reinforcing that the LINC00954-ORF polypeptide is indeed derived from lncRNA LINC00954. The polypeptide possesses its tumor-suppressor features to attenuate cell growth, migratory ability and invasiveness in A549 cells and its promoting activity to sensitize A549/PEM cells to PEM.

With the continuous discovery of polypeptides embedded in lncRNAs, unraveling their molecular processes has become an increasingly complex endeavor. Through mass spectrometry analysis, Huang and colleagues identified 485 proteins that interact with the lncRNA HOXB-AS3-encoded polypeptide and found that these interactors have relevance to RNA splicing (Huang et al. 2017). Zhu and colleagues found 170 protein interactors with polypeptide RBRP hidden in lncRNA LINC00266-1, with the majority of them being the RNA-binding proteins, and as a consequence, this polypeptide is validated to regulate m6A recognition (Zhu et al. 2020). Our results identified 1182 protein interactors with LINC00954-ORF polypeptide, which are related to diverse biological processes and pathways, such as DNA replication. These may contribute to the LINC00954-ORF polypeptide’s tumor-suppressor features in A549 LUAD cells. Future research should focus on these findings by elucidating the precise mechanisms underlying this polypeptide’s functions based on these identified interactors. The DNA-replication regulator PCNA and the cell-division inducer CDK1 are essential for cancer cell proliferation and tumorigenesis (González-Magaña and Blanco 2020; Tong et al. 2021). Our data demonstrated the inhibitory impact of the LINC00954-ORF polypeptide on the expression of the two factors, which suggests that this polypeptide may exert anti-growth effect in A549 and A549/PEM cells, at least in part, through the suppression of PCNA and CDK1. Further experiments will be conducted to elucidate the molecular processes through which the LINC00954-ORF polypeptide modulates the two factors.

Through TCGA-LUAD prognosis analysis, we found that high MIR4435-2HG level predicted poor prognosis of LUAD patients, and ADAMTS9-AS2 expression was positively associated with overall survival within 4000 days of post-operative follow-up. Elevated expression of MIR4435-2HG is related to poor prognosis and aggressive tumor behavior of various cancers including NSCLC, which might attribute to its ceRNA activity via miRNA competition and its regulatory impact in a variety of signaling pathways, such as TGF-β and β-catenin pathways (Zhong et al. 2022). Recent work indicates that reduced expression of ADAMTS9-AS2 is a biomarker for poorer prognosis in LUAD patients, which is found to be related to immune infiltrates (Lin et al. 2021). ADAMTS9-AS2 is downregulated in LUAD revealed by bioinformatics, and its interacted proteins have a close relationship with epithelial-to-mesenchymal transition (EMT) and Beta3 integrin cell surface interactions (Liu et al. 2021).The lncRNA/miRNA/mRNA ceRNA crosstalk mediated by ADAMTS9-AS2 is also predicted to be associated with LUAD prognosis (Zhao et al. 2021). Our results also revealed the association of high lncRNA LINC00954 expression with better outcomes in LUAD patients. Given that LINC00954 is a relatively unexplored lncRNA, not much information in available in public databases and published reports about its related factors in affecting prognosis of LUAD patients and its specific associations with pathological T stage, N stage, and pathological stage of LUAD tumors. Furthermore, the current exploration is limited by the lack of investigation in LINC00954’s function, which is warranted to perform in future work. Moreover, the in vivo biological functions of the LINC00954-ORF polypeptide have not been investigated, which will be addressed in future studies.

In summary, the LINC00954-ORF polypeptide embedded in lncRNA LINC00954 possesses tumor-suppressor features in A549 and PEM-resistant A549 cells and sensitizes PEM-resistant A549 cells to PEM. Our current findings provide evidence for the idea that the LINC00954-ORF polypeptide is a potential anti-cancer agent in LUAD.

## Supplementary Information

Below is the link to the electronic supplementary material.

Supplementary file1 (XLSX 28 KB)

Supplementary file2 (XLSX 71 KB)

Supplementary file3 (XLSX 9 KB)

Supplementary file4 (XLSX 13 KB)

## Data Availability

The data and material presented in this manuscript is available from the corresponding author on reasonable request.
